# Prediction of a Large-Scale Database of Collision Cross-Section and Retention Time Using Machine Learning to Reduce False Positive Annotations in Untargeted Metabolomics

**DOI:** 10.3390/metabo13020282

**Published:** 2023-02-15

**Authors:** Marie Lenski, Saïd Maallem, Gianni Zarcone, Guillaume Garçon, Jean-Marc Lo-Guidice, Sébastien Anthérieu, Delphine Allorge

**Affiliations:** 1 ULR 4483, IMPECS—IMPact de l’Environnement Chimique sur la Santé humaine, CHU Lille, Institut Pasteur de Lille, Université de Lille, F-59000 Lille, France; 2 CHU Lille, Unité Fonctionnelle de Toxicologie, F-59037 Lille, France

**Keywords:** mass spectrometry-based metabolomics, ion mobility-mass spectrometry, metabolomics data analysis, machine learning, collision cross-section, retention time

## Abstract

Metabolite identification in untargeted metabolomics is complex, with the risk of false positive annotations. This work aims to use machine learning to successively predict the retention time (Rt) and the collision cross-section (CCS) of an open-access database to accelerate the interpretation of metabolomic results. Standards of metabolites were tested using liquid chromatography coupled with high-resolution mass spectrometry. In CCSBase and QSRR predictor machine learning models, experimental results were used to generate predicted CCS and Rt of the Human Metabolome Database. From 542 standards, 266 and 301 compounds were detected in positive and negative electrospray ionization mode, respectively, corresponding to 380 different metabolites. CCS and Rt were then predicted using machine learning tools for almost 114,000 metabolites. R^2^ score of the linear regression between predicted and measured data achieved 0.938 and 0.898 for CCS and Rt, respectively, demonstrating the models’ reliability. A CCS and Rt index filter of mean error ± 2 standard deviations could remove most misidentifications. Its application to data generated from a toxicology study on tobacco cigarettes reduced hits by 76%. Regarding the volume of data produced by metabolomics, the practical workflow provided allows for the implementation of valuable large-scale databases to improve the biological interpretation of metabolomics data.

## 1. Introduction

The metabolome of a biological system is influenced by physiological, pathological, or environmental conditions [1]. As it gathers the final products of the cellular process, the exhaustive measurement of metabolite changes by metabolomics provides dynamic and sensitive information closely linked to its phenotype. Investigating the metabolome allows the identification of metabolic fingerprints that can then be used as biomarkers and/or provides new mechanistic perspectives leading to a particular phenotype [2,3]. Recent developments in mass spectrometry (MS) technology, informatics, and analytical chemistry have made it possible to comprehensively analyze the metabolome [4,5] with a high level of sensitivity [6] compared to nuclear magnetic resonance-based metabolomics. Additionally, high throughput analyses like high-resolution MS in full scan mode allow the rapid achievement of large-scale studies of hundreds of compounds (untargeted metabolomics), which is an evident benefit over analyses that target a restricted number of metabolites or pathways (targeted metabolomics).

Untargeted metabolomics is a multistep process involving first sample collection, preparation, and analysis that generates data, then data processing and chemometrics that generate a candidate list of features, and finally, metabolite identification [7]. This final step gives biological meaning to MS data [8]. A consensus by the Chemical Analysis Working Group of the Metabolomics Standards Initiative (MSI) reported different levels of confidence in the annotation depending on the method of identification used [9]. A confident and definitive identification (level 1) is hit when two or more orthogonal properties fit with data from authentic standard compounds in identical analytical settings. When the latter are unavailable, a comparison of experimental data with public libraries could lead to a putative compound annotation (level 2) or class annotation (level 3). Finally, unknown features discriminated with spectral data are classified at the lowest confidence level (level 4). Therefore, feature annotation is achieved by comparing experimental measurements to existing in-house or external databases of known metabolites to generate potential candidates [10]. Several commercial or open-source databases containing spectral data in libraries (Human Metabolome Database (HMDB) [11], Metlin [12]…) were developed and are continuously updated by the scientific community. However, confident and unequivocal structure identification could quickly be an issue when a candidate is not found (limited number of spectra) or when several candidates are proposed (false positives), increasing the probability of misidentification [13,14]. Therefore, it becomes important to use other readily obtained physicochemical properties for better metabolite identification.

Ion mobility-mass spectrometry (IMS-MS) is a fast two-dimensional separation of ions based on their mobility in a buffer gas. Importantly, this mobility is structure-dependent and is not affected by equipment or experimental factors (matrix effects, variations in mobile phase composition, and chromatography settings, ionization mode, acquisition settings…), unlike retention time (Rt) and mass spectrum [15], resulting in a high degree of repeatability and therefore facilitating database queries [16]. The physical property measured in IMS-MS is the collision cross-section (CCS). Thus, it provides the orthogonal separation to improve signal-to-noise, resolution, and isomeric metabolite separation [17], participating in the reduction of misidentification. However, the favorable contribution of CCS is currently limited by the poor availability of CCS reference values [18,19,20,21,22]. New experimental and computational approaches to predict those parameters for a large number of compounds is highly valuable. Several studies have developed, or applied machine learning-based prediction approaches [23]. Softwares like AllCCS [22], CCS Predictor [24], DeepCCS [25], MetCCS Predictor [26], or LipidCCS predictor [27] can efficiently generate a model when molecular descriptors are provided [15]. Molecular descriptors are numeric information generated by mathematical treatment of compound structures that characterize the physico–chemical properties of metabolites (ex: polarity, LogP…) [28]. In contrast, CSSBase is a web interface (https://CCSbase.net) (accessed on 6 May 2022) that provides access to a ready-to-use predictive model, allowing rapid prediction of CCS values directly from SMILES structures (Simplified Molecular Input Line Entry System representation), using a cluster-based prediction model [29]. This platform allows a broad coverage of chemical structure diversity and can thus be easily used in existing metabolomics workflows.

The Rt of a compound is defined by its chemical interactions with the chosen mobile phase and stationary phase. Metabolite retention can be improved by optimizing solvent gradient elution, nature, and dimensions of the chromatographic column or chromatographic settings [30]. Rt is often decisive in feature annotation but usually relies on the availability of authentic chemical standards that are applied to experimental conditions. In untargeted metabolomics, the transferability of Rt database between laboratories is not achievable because of the absence of standardized assays across different laboratories. Multiple machine learning models for the prediction of Rt have already been described, including quantitative structure–retention relationship (QSRR) models [31,32,33,34,35]. QSRR strategies have been used to accelerate the method development process by comparing predicted separation with different columns [36] or to enhance the confidence of identifications [37]. Software packages, such as the QSRR Automator [38], exist to automate Rt prediction model creation. Structure and chromatographic data from known metabolites, obtained from their SMILES and from chemical standards analyzed using a particular LC method, are used to generate a model. It identifies relations between chromatographic retention and the molecular descriptors, theoretically allowing to predict Rt for any metabolite whose molecular descriptors can be calculated [39].

In the present work, we aim to describe the workflow permitting the generation of a large-scale in-house database of Rt and CCS predicted with published machine learning models. Integration of these data with other sources of information, such as accurate mass, MSe fragmentation, and isotope pattern for facilitating the identification of compounds, is illustrated in an application to toxicology data.

## 2. Materials and Methods

### 2.1. Chemicals and Standards

Solutions used were: acetonitrile (UPLC-MS grade, Waters, Milford, MA, USA), methanol (UPLC-MS grade, Waters), Milli-Q purified water (Millipore, Burlington, MA, USA), formic acid (UPLC-MS grade, Honeywell, Charlotte, NC, USA), ammonium formate (Reagent-grade, Sigma-Aldrich, St. Louis, MO, USA), and chloroform (VWR Chemicals, Radnor, PA, USA). Chemical standards (MSMLS) were purchased from Sigma-Aldrich. This library was chosen for the broad chemical and functional diversity of metabolites included. It contains 634 standard metabolites sampled into seven 96-well plates at 5 µg per well, including 37 duplicates. An associated spreadsheet with information, such as metabolite identification, molecular formula, and SMILES was used to build our targeted database. The compounds were dissolved using two different solutions (5% methanol for plates 1–5 and chloroform:methanol:water 1:1:0.3 for plates 6–7) following the manufacturer’s instructions to obtain a 20 µg mL^−1^ concentration. Stock solutions were pooled with a maximum of 12 compounds to obtain 56 solutions at 1.6 µg mL^−1^ to perform simple multiplex injections for LC-MS analysis.

### 2.2. LC-MS Conditions

Analyses were conducted on a liquid chromatograph system coupled to high-resolution mass spectrometry (LC-HRMS). Chromatographic separation was obtained with the following characteristics: Instrument: Acquity UPLC I-Class system (Waters); column: Acquity UPLC HSS T3 (1.8 μm, 150 × 2.1 mm; Waters); column temperature: 50 °C; flow rate: 0.4 mL min^−1^; autosampler temperature: 10 °C; volume of injection: 15 μL. Separation was performed in a gradient elution mode. Mobile phases for the multistep gradient in the positive mode were solution A: aqueous solution of ammonium formate (3 mM) with 0.1% formic acid and solution B: acetonitrile with 0.1% formic acid (*v*/*v*). The elution gradient was: 100% A for 1 min, 0–1% B for 1 min, 1–3% B for 2 min, 3–99% B for 13 min, 99% B for 3 min, 99–0% B for 0.5 min, and 100% A for 2.5 min. Detection was performed on a Vion IMS-QToF mass spectrometer (Waters) with the following settings: ionization source: electrospray operating in positive (ESI+) and negative (ESI-) modes; source temperature: 120 °C; desolvation temperature: 600 °C; cone gas flow: 50 L h^−1^; desolvation gas flow: 1000 L h^−1^; capillary voltage: 0.5 kV in ESI+ or 2 kV in ESI-; *m/z* range: 50–1000; scan time: 0.25 s; lock mass reference: leucine enkephalin (*m*/*z* 556.2766) solution at 200 ng mL^−1^; infusion intervals: 5 min; acquisition mode: high-definition MSe; low collision energy: 6 V; high collision energy ramp: 14–56 V; IMS drift gas and collision gas: nitrogen; ion mobility and mass calibrations solution: Major Mix IMS/ToF Calibration Kit (Waters). These parameters allow for achieving a mass resolving power of >20,000 FWHM.

Data analysis of the mixes was semi-automatically performed through the Unifi software (version 1.9.4.053 Waters MS Technologies, Manchester, UK) to obtain the Rt, response, and CCS of the standards after manual verification of the peak integration. Adducts considered were [M+H]+, [M+K]+, [M+Na]+, [M+Cl]−, [M+HCOO]−, and [M+CH3OO]−. Accurate mass, Rt, CCS, and fragmentation patterns were used to build a targeted database of LC and MS properties.

### 2.3. CCS Prediction

CCSBase is an electronic interface (https://CCSbase.net) (accessed on 6 May 2022) for accessing the CCS predictive model [29]. It calculates the predictive CCS values of adducts using SMILES. Original performances of CCSBase were described by Ross et al. [29] with an R^2^ score, a mean absolute error, and a root mean squared error at 0.991, 3.83 Å^2^, and 5.48 Å^2^, respectively. In the study, all adducts considered by CCSBase, namely [M+H]+, [M+K]+, [M+Na]+, [M+Na-2H]−, [M+NH4]+, [M]+, [M-H]−, and [M]−, were taken in account. A batch prediction was performed for a dataset of metabolite structures freely available in HMDB v4.0 [11], which gathers up to 114,000 human metabolites, covering the majority of untargeted metabolomic data sets. A linear regression was performed comparing predicted and measured CCS of adducts from standard compounds. Measured CCS were included in this comparison if the standard were listed in HMDB, and if they presented common adducts with predictions. The coefficient of determination R^2^ between the predicted and the experimental CCS data and mean absolute error permitted to evaluate the model. The best fit of linear regression was calculated, with an interval of +/− 2 standard deviations (SD). All statistical analyses and figure production of this manuscript were conducted under R language and environment [40].

### 2.4. Rt Prediction of Small Molecules

QSRR Automator [38] builds regression retention models. Based on their SMILES, chemical structures were converted into their numerical representation by expressing them through structural descriptors produced by informatic algorithms of QSRR Automator. First, using a defined training data set, the machine learning algorithm learns the “rule” between molecular descriptors and their experimental Rt values to establish prediction models and select the best model. QSRR algorithm identifies descriptors that positively impact model performance. Selection and optimization of regression algorithms were carried out by automated procedures and evaluated thanks to the R^2^ score and mean absolute error. Then, the external validation data set is used to validate and evaluate the prediction error. Cross-validation (*n* = 10) provides an estimate of the accuracy of the Rt prediction for compounds that were not used in its development or optimization, evaluated thanks to the R^2^ score, mean absolute error, and SD. Once a valid model was selected, Rt predictions were performed for metabolites from the HMDB v4.0. The best fit of linear regression was calculated with an interval of +/− 2 SD.

### 2.5. Reduction of the Occurrence of False Positive Annotations in Untargeted Metabolomics: Application to Toxicology Data

We analyzed LC-HRMS data from an ongoing study assessing the potential toxicity of tobacco cigarette fumes on human bronchial epithelial BEAS-2B cells to demonstrate the relevance of the predicted large-scale database of collision cross-section and retention time to metabolomics. The exposure protocol was adapted from Dusautoir et al. [41]. Briefly, BEAS-2B cells cultured at air–liquid interface was exposed to four puffs of tobacco cigarette emissions or to sterile air (negative control) in four replicates per exposure. Twenty-four hours after exposure, cell metabolism was quenched by the addition of ice-cold methanol:water (80:20, *v*/*v*) mixture. Cells were harvested using a cell scraper. Deproteinization was performed by adding the same methanolic mixture, vortexing, and centrifuging at 14,000× *g* at +4 °C for 15 min. Supernatants were concentrated to dryness with speedvac and reconstituted before injection in a water:methanol (90:10, *v*/*v*) mixture. After metabolomic analyses, LC-HRMS data were analyzed with Progenesis QI (Nonlinear Dynamics, UK) for feature extraction. Data normalization and statistical analyses were conducted under the R environment [40] on the features detected in ESI+ and ESI−. When searching against HMDB, two identification strategies were evaluated: (1) with an *m*/*z* (tolerance set at 5 mDa), isotope and fragmentation match only, and (2) with an *m*/*z*, isotope, fragmentation, CCS, and Rt match of the created predicted large-scale database.

## 3. Results

### 3.1. Analysis of Standard Compounds and Generation of an In-House Database

**Figure 1:**
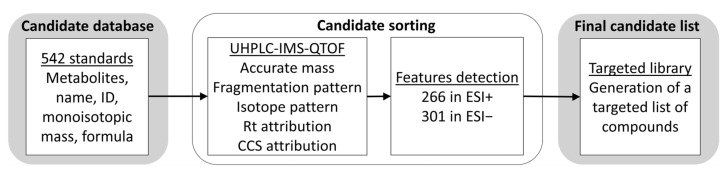
Workflow and results of the targeted-based metabolomic approach developed.

A total of 542 standards were originally used in the candidate database. Each set of data was manually examined for errors. Figure 1 describes the workflow used for the targeted database construction. The candidate sorting step has allowed the detection of 266 and 301 compounds in ESI+ or ESI−, respectively, corresponding to a total of 380 different metabolites. Accurate mass, Rt, CCS, and fragmentation were used to build a targeted database of LC and MS properties.

**Figure 2:**
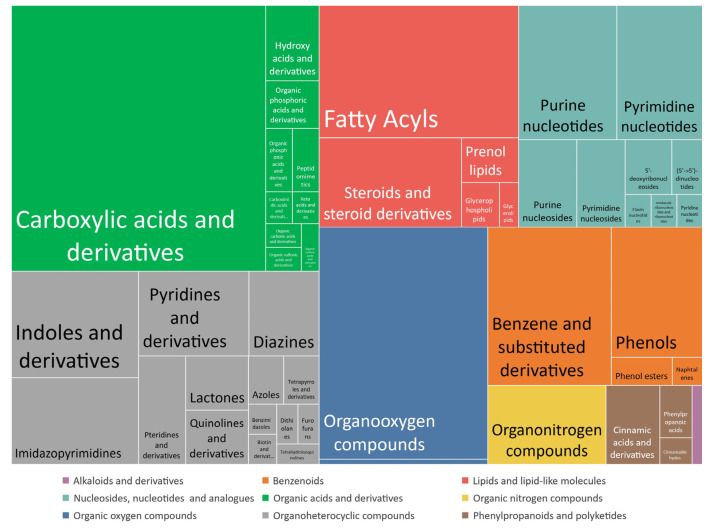
Chemical taxonomy of metabolites included in the targeted library and proportion of each superclass and class.

Metabolites belong to different chemical classes and are mainly carbohydrates, carboxylic acids, lipids, nucleotides, and organoheterocyclic compounds (Figure 2) involved in different pathways that reflect the metabolic status in biological matrices of interest.

### 3.2. CCS Prediction and Creation of a CCS Database

**Figure 3:**
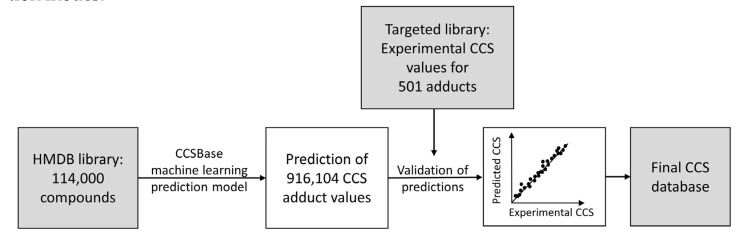
Workflow of CCS prediction.

The CCS database was generated according to the workflow described in Figure 3. Predictions were performed for almost 114,000 metabolites from the HMDB v4.0 database, generating 916,104 CCS adduct values. Results were validated with a validation set composed of 501 measured CCS adduct values from 297 standard compounds in both ionization modes.

**Figure 4:**
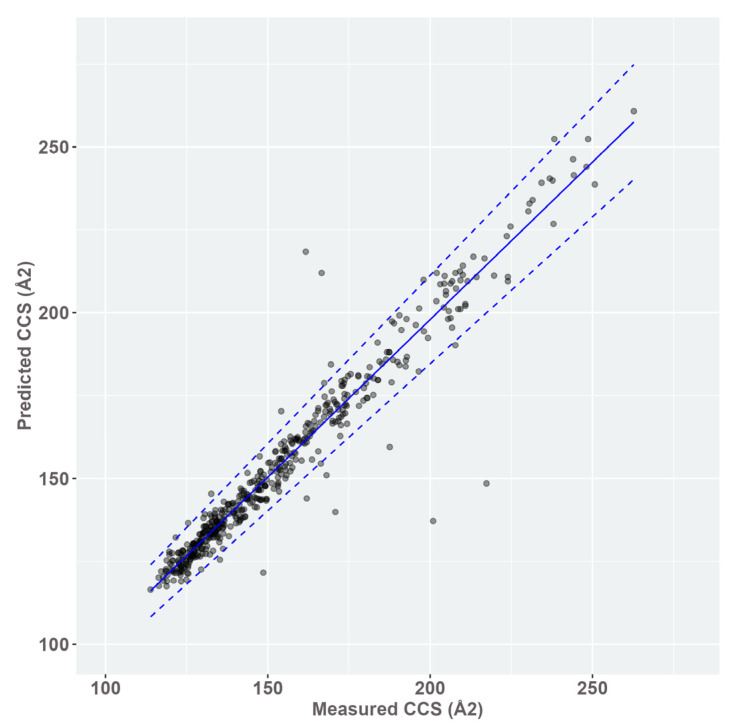
Predicted CCS (Å^2^) using CCSBase algorithm [29] by measured CCS (Å^2^). Validation was performed on the CCS of 501 adducts from 297 standard compounds measured in ESI− and ESI+. Linear regression: adjusted R^2^ = 0.938. Blue line: best fit of linear regression. Dashed blue lines best fit +/− 6.72% (2 SD). Predicted CCS = 0.95 × measured CCS + 7.92.

Figure 4 illustrates the match between predicted and experimentally determined CCS. Outliers were kept as part of the data set in the absence of any evidence that they were the result of an error. The R^2^ score of the linear regression achieves 0.938 and the mean absolute error was calculated at 3.94 Å^2^, while the SD reaches 6.11 Å^2^ or 3.36%. The predicted CCS = 0.95 × measured CCS + 7.92. The resulting output table of CCS allowed us to build our large-scale in-house reference database.

### 3.3. Rt Prediction and Creation of an Rt Database

**Figure 5:**
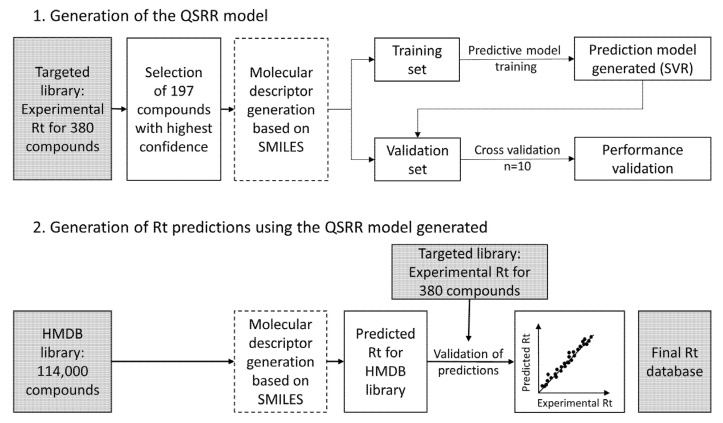
Workflow of Rt prediction.

The Rt prediction workflow is described in Figure 5. In total, 204 compounds from the developed method were selected for the QSRR model; 114 were detected in both ESI+ and ESI−, while 90 were detected only in one ionization mode (45 for each ionization mode). Seven compounds were excluded due to incomplete data in molecular descriptors. Support vector regression (SVR) algorithm based on 113 molecular descriptors presented the best performances, with the R^2^ score at 0.999 and the mean absolute error at 0.10 min for the training set. The validation set tested by cross-validation (*n* = 10) validated the model with the following performances: mean of cross-validation R^2^ score 0.898, mean absolute error 0.81 min, and standard deviation of the mean absolute error 0.15 min. Detailed results are presented in Table S1 and Figure S1. Rt predictions were performed for almost 114,000 metabolites from HMDB v4.0. The resulting output table of Rt allowed us to build our large-scale in-house reference database.

### 3.4. Reduction of the Occurrence of False Positive Annotations in Untargeted Metabolomics: Application to Toxicology Data

**Figure 6:**
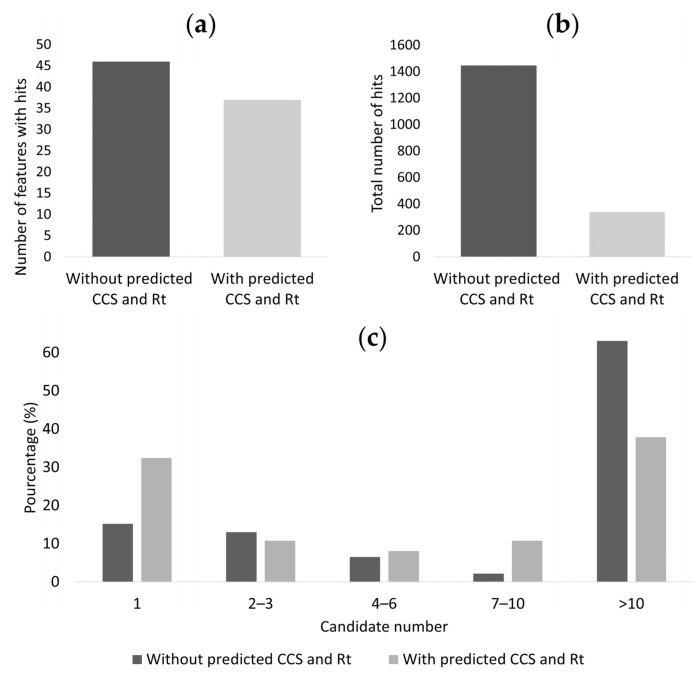
Contribution of the predicted CCS and Rt database to metabolite identification in untargeted metabolomics applied to toxicology. From the two match methods: (**a**) number of features with hits, (**b**) total number of hits (**c**), percentage distribution of features with different metabolite hits.

We assessed the potential toxicity associated to tobacco cigarette fumes on the human bronchial epithelial BEAS-2B cells using metabolomics. Among the 3591 features detected in ESI+ and ESI−, 51 features were significantly deregulated by cigarette smoke compared to controls and needed to be identified. As illustrated in Figure 6a, 46 out of 51 features had one hit or more (90%). The number of hits exceeded 10 hits for the major part of the features. For the method combining *m*/*z*, CCS, and Rt match search, CCS and Rt match tolerances were set at 16 Å^2^ and 1.1 minutes, respectively, according to the determined CCS and Rt index filter expressed as mean error +/− 2 SD. Only 37 out of 51 features (72%) had one or more metabolite hits. Seventy-six percent of hits were filtered using the predicted large-scale database (Figure 6b). The percentage of features with only one hit significantly increased with the additional CCS and Rt match (+53%), while the percentage of features with more than 10 hits decreased in the same conditions (−39%) (Figure 6c). For further identification, possible candidates for each compound are ranked by Progenesis QI on an overall score based on the *m*/*z* match, isotope similarity, fragmentation score, CCS, and Rt error (data not shown).

## 4. Discussion

LC-HRMS is an uncontestably powerful analytical approach employed in both targeted and untargeted metabolomics. We used an UPLC-IMS-QTOF to create an accurate in-house database using a commercial library of metabolite standards. Based on experimental results, we used two existing prediction models (CCSBase and QSRR Automator) to predict CCS and Rt values of a large-scale database to increase confidence in metabolite annotation. Associating Rt and CCS is relevant as it can provide complementary information coming from chromatographic and ion mobility separation or even replace other orthogonal properties (isotope similarity and fragmentation score) for putative compound annotation. Moreover, for all metabolites, predictions were performed for protonated and deprotonated ions as well as adducts, each having the same Rt but a different CCS [42]. The co-occurrence of adducts is common when analyzing heterogeneous biological samples [43]. Gathering predictions for multiple metabolic features of the same metabolite is valuable information, allowing for cross-validation of identification. The predicted database is presented in Table S2.

The comparison of mass spectra, Rt, CCS, and accurate mass of a feature with experimental data acquired from standard compounds measured under the same analytical settings permits achieving the highest level of identification confidence. An in-house database of 380 different metabolites was generated, allowing a confident and definitive identification (level 1 according to the MSI [9]). Metabolites were separated using an Acquity UPLC HSS T3 column that possesses superior polar-compound retention and aqueous mobile phase compatibility compared to more classical stationary phases. It could be used for the retention of mid-polar to apolar analytes. The Sigma library used contains several polar metabolites that cannot be retained in those analytical conditions, explaining the lack of detection for some metabolites [44]. Moreover, with up to 114,000 chemicals deposited in HMDB, only a few percent of these compounds could be covered with authentic standards. Therefore, structure identification in untargeted metabolomics analyses remains a significant challenge. By predicting chromatographic Rt and CCS from experimentally acquired data, this targeted library represents a starting point to potentially give access to a detailed sample composition for future untargeted metabolomic studies. Using this methodology, we were able to drastically expand the number of metabolites at level 2 or level 3 annotations [9].

Predictions using machine learning are data-driven approaches providing predictions for metabolites with corresponding properties [45]. After the training of the model, predictions could be generated immediately for other compounds.

For CCS predictions, CCSBase is a machine learning-based prediction model built from a combined database, enabling to cover an important variety of structural compounds, participating in the transferability of this model. Indeed, large-scale CCS predictions were validated with our experimental data with a low bias and a high R^2^ at 0.938. A CCS index filter defined as mean error +/− 2 SD, i.e., maximum 16.16 Å^2^, could be used as the threshold for excluding false positives. This match tolerance, reflecting the deviation of analytes or family of analytes or type of adducts, is relatively large compared to other work demonstrating that median relative errors as low as 3 to 5% are reachable using other models [22,24,25,26,27]. However, excluding false positive identifications with a CCS match higher than the defined threshold remains of great importance when considering the number of possible matches when using m/z match, isotope similarity, and fragmentation score only. Moreover, this additional separation process participates in better detection of compounds presenting contaminant mass spectra due to the co-elution or a poor abundance.

For Rt predictions, the workflow was different as we trained and validated an accurate machine-learning model based on compounds with various physicochemical properties. The training set allowed the model to be trained, while the test set made of unknown data for the trained model allowed the model to be validated. With this strategy, the model was estimated with small error differences in favour of minimum overfitting. An Rt index filter defined as mean error +/− 2 SD, i.e., maximum 1.11 min, could be used as a threshold for eliminating the majority of misidentified compounds. Outliers could be due to software bias, random noise in the data used or errors in the attribution of standards. Naylor et al. described the QSRR Automator’s original performances on various chromatographic columns. They showed errors in predictions within 1 min for the majority of predictions, and within 2 min for almost all predictions [38]. QSRR here performs comparably to previously published methods [34,36]. In relatively short run time methods, as in our method, many metabolites have very close Rt, including isomers with Rt that fall within 10 s of each other. Our database does not permit the distinction between those metabolites but is adequate to differentiate between clearly separated compounds of the same mass and reduce false positive identifications, leading to an advanced biological interpretation of results. Even if the generated model was based on compounds with various physicochemical properties separated and identified with an optimized method, particular attention should be dedicated to avoiding inaccurate results, including (i) compounds not retained in the column (ii) compounds retained after the observed Rt of the training data (iii) compounds with physicochemical properties that differ from the training set. For example, the in-house database presented here was generated from a large variety of chemical standards dedicated to metabolomics analyses but did not include complex high molecular weight compounds. Biased predictions for those metabolites should be excluded. The Rt database that we created is strictly related to our chromatographic conditions, so it can be directly useful only for those who decide to strictly adapt our choice of column, mobile phases, and flow rates. In reversed-phase chromatography, authors suggested that Rt from a defined method can be projected in other chromatographic settings as soon as the elution order of metabolites is preserved [46]. Most of the time, laboratories employ a distinct chromatographic setting depending on the separation required. We here presented a practical workflow, with the objective of generating QSRR models and predictions for every set of LC conditions.

Some limitations of the present study must be mentioned. The number of compounds is relatively small, resulting in a limited number of experimental data that could influence the performance of models. However, this limitation is counterbalanced by the quality of data since we included data from authentic standards with the highest confidence possible. The resulting performances could have been further validated by performing a side-by-side comparison with other existing machine-learning tools. Such a comparison has already been described elsewhere [22,24,25,26,27]. Instead of that, the chosen strategy consisted of emphasizing the usefulness of our workflow with concrete application on biological data. Finally, by associating Rt predictions with CCS predictions, the generated large-scale database is strictly related to the instrumental configuration, but the workflow could be largely generated to other experimental conditions.

Most prediction models or workflows previously reported discuss one or the other predicted property (CCS or Rt), while only a few associate multi-dimensional information for metabolite annotation [47,48,49,50]. Interestingly, all of them are dedicated to lipids or exogenous compounds, while our workflow predicted a database including small molecules found in the human body, including water-soluble or lipid-soluble endogenous metabolites and exogenous compounds. Regarding the tremendous interest of the scientific community in metabolomics, providing a practical workflow is of large importance for analytical chemists or biologists who cannot develop machine learning models but who want to improve the biological interpretation of their metabolomics data. The usefulness of our combined large-scale predicted database was demonstrated with an application of biological data generated from a toxicology study on tobacco cigarettes. The results demonstrated that the introduction of CCS and Rt values for metabolite identification could significantly reduce false positive identifications, with the benefit of narrowing the search scope and improving the identification accuracy.

## 5. Conclusions

In this study, a workflow was introduced to remove false positive annotations in nontargeted metabolomics studies. The procedure includes the implementation of a combined CCS and Rt-restricted database starting from a commercial library of metabolite standards. This experimental database has been used to predict CCS and Rt of a large-scale dataset using existing machine learning tools. As illustrated by an application on a metabolomic study on tobacco cigarette toxicity, the presented workflow reduces the occurrence of false positive annotations in untargeted metabolomics and adds confidence to the identification of metabolites. This database has been integrated into the protocol used in our laboratory for untargeted metabolomics analyses and is freely downloadable. When making the assumption that the created database could be a representative subset of compounds present in the human metabolome, biological interpretation of metabolomics data is notably improved, giving new insights into biomarker research or mechanisms that generate a specific phenotype. We suggest using our data as a methodological starting point for the development of a large-scale in-house reference database based on artificial intelligence tools, providing a practical and effective workflow to improve the predictive confidence of metabolomic studies at a large-scale level.

## Data Availability

The data presented in this study are available on request from the corresponding author. The data are not publicly available due to privacy.

## Supplementary Materials

The following supporting information can be downloaded at: https://www.mdpi.com/article/10.3390/metabo13020282/s1, Figure S1: Performances of the SVR model using QSRR Automator algorithm; Table S1: Linear regression of Predicted Rt (min) using QSRR Automator algorithm by measured Rt (min) of compounds used from the developed method. This figure was generated by QSRR Automator from all the data available for model construction (including training data); Table S2: Large-scale in-house reference database.

## Abbreviations

CCS	collision cross-section
ESI	electrospray ionization
HMDB	human metabolome database
HRMS	high-resolution mass spectrometry
IMS	ion mobility spectrometry
LC	liquid chromatography
*m/z*	mass to charge ratio
MS	mass spectrometry
MSI	metabolomics standards initiative
MSMLS	mass spectrometry metabolite library of standards (Sigma-Aldrich)
QSRR	quantitative structure retention relationships
QTOf	quadrupole time-of-flight
Rt	retention time
SD	standard deviation
SMILES	simplified molecular input line entry system
SVR	support vector regression
UHPLC	ultra high-performance liquid chromatography
