# Supplementary material for: Prediction of a Large-Scale Database of Collision Cross-Section and Retention Time Using Machine Learning to Reduce False Positive Annotations in Untargeted Metabolomics
Source: Metabolites. 2023 Feb 15;13(2):282. doi: 10.3390/metabo13020282 (PMC9962007; doi:10.3390/metabo13020282)

SUPPLEMENTARY MATERIALS

Table S1. Performances of the SVR model using QSRR Automator algorithm.

Scoring Metric	Value(s)
Final Model R2 Score	0.999
Final Model Mean Absolute Error	0.100
Cross Validation R2 Values	[0.917 0.938 0.849 0.890 0.884 0.843 0.909 0.872 0.949 0.926]
Mean of Cross Validation R2 Values	0.898
Median of Cross Validation R2 Values	0.900
Standard Deviation of Cross Validation R2 Values	0.034
Cross Validation Mean Absolute Error Values	[0.933, 0.649, 0.761, 0.948, 1.035, 1.059, 0.666, 0.752, 0.661, 0.676]
Mean of Cross Validation Mean Absolute Error Values	0.814
Median of Cross Validation Mean Absolute Error Values	0.756
Standard Deviation of Cross Validation Mean Absolute Error Values	0.154

Figure S1. Linear regression of Predicted Rt (min) using QSRR Automator algorithm by measured Rt (min) of compounds used from the developed method. This figure was generated by QSRR Automator from all the data available for model construction (including training data).

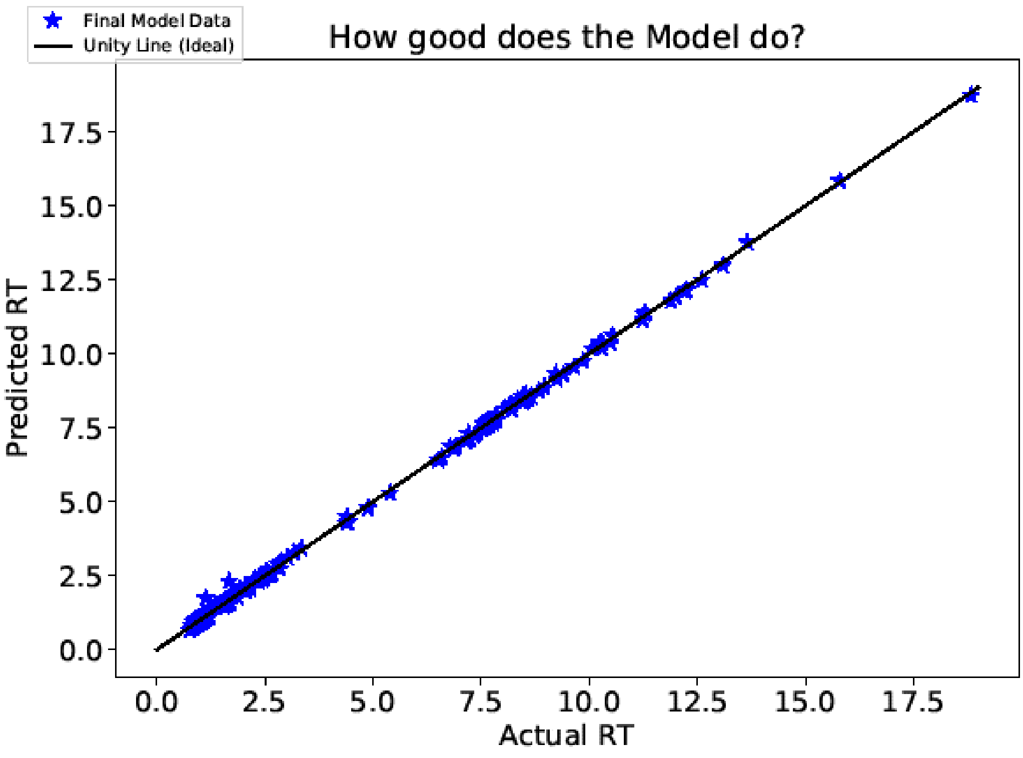

Supplement: Supplementary file 1 [file metabolites-13-00282-s001.zip › Supplementary material_V2.pdf]
